# Compensatory plasticity: time matters

**DOI:** 10.3389/fnhum.2014.00340

**Published:** 2014-06-12

**Authors:** Latifa Lazzouni, Franco Lepore

**Affiliations:** Département de Psychologie, Centre de Recherche en Neuropsychologie et Cognition, Université de MontréalMontréal, QC, Canada

**Keywords:** compensatory plasticity, onset time, blindfolding, cortico-cortical pathways

## Abstract

Plasticity in the human and animal brain is the rule, the base for development, and the way to deal effectively with the environment for making the most efficient use of all the senses. When the brain is deprived of one sensory modality, plasticity becomes compensatory: the exception that invalidates the general loss hypothesis giving the opportunity of effective change. Sensory deprivation comes with massive alterations in brain structure and function, behavioral outcomes, and neural interactions. Blind individuals do as good as the sighted and even more, show superior abilities in auditory, tactile and olfactory processing. This behavioral enhancement is accompanied with changes in occipital cortex function, where visual areas at different levels become responsive to non-visual information. The intact senses are in general used more efficiently in the blind but are also used more exclusively. New findings are disentangling these two aspects of compensatory plasticity. What is due to visual deprivation and what is dependent on the extended use of spared modalities? The latter seems to contribute highly to compensatory changes in the congenitally blind. Short-term deprivation through the use of blindfolds shows that cortical excitability of the visual cortex is likely to show rapid modulatory changes after few minutes of light deprivation and therefore changes are possible in adulthood. However, reorganization remains more pronounced in the congenitally blind. Cortico-cortical pathways between visual areas and the areas of preserved sensory modalities are inhibited in the presence of vision, but are unmasked after loss of vision or blindfolding as a mechanism likely to drive cross-modal information to the deafferented visual cortex. The development of specialized higher order visual pathways independently from early sensory experience is likely to preserve their function and switch to the intact modalities. Plasticity in the blind is also accompanied with neurochemical and morphological changes; both intrinsic connectivity and functional coupling at rest are altered but are likewise dependent on different sensory experience and training.

## Introduction

If visual deprivation happens within the critical period of brain development early in life, it deeply affects the normal function of the brain. The neural system has the capacity to adapt to the loss of one modality by undergoing plastic changes in its structure, connectivity, function and neural interactions. Such “compensatory plasticity” takes place within the visual cortex that responds to cross-modal auditory and tactile stimulations. Changes to the deprived visual cortex spread also to higher visual areas, which project to it. For decades, researchers investigated functional reorganization after visual loss by studying intact auditory and tactile modalities; these remaining senses are very important to the blind, as they constitute the only way to interact effectively with their environment in functions such as sound processing and localization used in spatial navigation and tactile processing for object recognition and Braille reading. Subsequent research also showed that blind individuals perform better in sensory and cognitive tasks both at behavioral level and in task-specific functional activations of occipital cortex to non-visual stimulation.

Blindness offers a natural model to assess effects of visual deprivation on the development of the human brain. Do blind individuals systematically outperform their sighted counterparts in sensory and cognitive processes? Is there a correlation between the superiority of the blind and visual areas recruitment? What are the neuronal mechanisms that underlie functional reorganization in light of recent research findings? What are the differences between early (congenital, before age of 5) and late (after age 14) loss of vision?

Here we review recent research on functional reorganization following long- and short-term visual deprivation through early and late blindness, and in blindfolded sighted individuals.

## Reorganization in the visually deprived brain

The general loss hypothesis (Rauschecker, 1995a,b) predicts that early sensory deprivation and the lack of visual experience during postnatal development of the brain induces a generalized degradation of sensory functions. Accordingly, blind individuals would be impaired in the calibration of their other senses and therefore be unable to form a map of the surrounding space. However, most findings of the last decades favor the contrasting hypothesis of compensatory adaptation. Here, the brain deprived of one sensory modality shows massive reorganization and adapts to sensory loss by developing superior abilities and recruits deafferented cortex to process information from preserved modalities (Lessard et al., 1998; Leclerc et al., 2000; Weeks et al., 2000; Voss et al., 2004).

Animal studies on dark reared (in the absence of light) or enucleated animals indicate plastic changes both behaviorally and physiologically (Rauschecker, 1995a,b; Maurer et al., 2005) Early visual deprivation in animals drives non-visual information to the visual cortex (Karlen et al., 2006; Piche et al., 2007). These studies helped put into light the anatomical connections that lay the ground on which non-visual inputs find their way to the deafferented visual cortex. In non-human primates, primary visual areas receive direct projections from the auditory cortex, and both V1 and V2 receive projections from parietal association areas (Falchier et al., 2002; Rockland and Ojima, 2003). In early blind individuals, auditory inputs are rerouted to the visual cortex rendering visual neurons responsive to auditory information—rewiring has taken place. This change in neuron characteristic function suggests the potential for adaptation and the importance of thalamic pathways in shaping the development and function of sensory cortices.

In humans, when a brain area suffers a lesion, the subsequent alteration of cortical function helps to better understand the role of this particular area in the normal brain. Hence, a proficient congenitally blind Braille reader suffered a lesion in bilateral occipital cortex after a stroke. She was no longer able to read Braille but was still capable of correct tactile processing (Hamilton et al., 2000). In this patient’s case the visual cortex before the stroke was involved in Braille reading, a cognitive process relying on tactile perception. As with prior studies (Sadato et al., 1996; Cohen et al., 1997; Büchel et al., 1998), the involvement of visual cortex in a higher order cognitive process was demonstrated in a blind person.

Short-term sensory deprivation using blindfolds gave additional insights into the neural mechanisms of brain plasticity. Performance, sensory thresholds and visual cortex activations to non-visual stimulation rapidly changed when participants’ vision was inhibited by masking (Kauffman et al., 2002; Merabet et al., 2008; Lazzouni et al., 2012). Further comprehension of plasticity stems from brain stimulation techniques such as transcranial magnetic stimulation (TMS), which modulate excitability of cortical neurons and the possibility to induce transient virtual lesions: TMS applied on the visual cortex of the blind resulted in a distortion of tactile perception and impaired ability to identify Braille letters. TMS applied to the visual cortex of sighted controls, however, had no effect on the performance in a tactile task (Cohen et al., 1997).

Does functional specialization of higher order areas involved in spatial motion processing and object recognition rely on early sensory experience, or does it develop independently from sensory modality? Early onset blindness seems to be crucial to preserve functional specialization of heteromodal visual areas and their shift to intact modalities. In early visual deprivation dorsal and ventral visual streams are preserved but adapted their modality to auditory and tactile. We can find a confirmation of such involvement of heteromodal areas in the activation of the visual motion area V5/MT by auditory and tactile motion (Poirier et al., 2006), as well as the activation of the lateral occipital complex in auditory processing using a vision substitution device (Amedi et al., 2007). Congenitally blind show activation of the right occipital gyrus and the cuneus in response to auditory motion processing which is yet another indication of the preservation of the dorsal stream in the blind to process auditory spatial information (Renier et al., 2010, 2014; Collignon et al., 2011). Further, recruitment of the right occipital cortex in odor discrimination and categorization supports the notion of heteromodal visual areas with modality independent specialization (Renier et al., 2013). The visual word form area is another example how a region maintains its functional specialization and processes letters via a visual-to-auditory-sensory substitution device in the blind (Arno et al., 2001; Poirier et al., 2006; Striem-Amit et al., 2012a,b).

This latter study raises an important question: Does super performance in the blind hold for any auditory, tactile processing and after training with substitutions devices? Actually, early and/or late blind individuals do not always outperform sighted controls on all auditory or tactile tasks. Many studies show a subgroup of early blind individuals and late blind with equal performance compared to controls when they localize for example monaural sounds on the horizontal plane (Lessard et al., 1998). Early blind have equal thresholds performance in tactile-visual acuity task of the tongue using a vision substitution device (Chebat et al., 2007b). When early and late blind show equal performance in sound localization compared to controls this coincides with the absence of occipital recruitment (Gougoux et al., 2005). Given particular experimental conditions, blind individuals have poor performances when they have to localize sounds in a complex acoustic environment. They probably fail to extract for example spectral cues for sounds presented in vertical elevation positions (Lewald, 2002; Kupers and Ptito, 2014).

## Superior auditory skills: developmental or use-dependent plasticity?

Blind individuals adapt to the loss of vision, are able to navigate effectively in close and far space and find their way nearly as safely as the sighted. To achieve this, they rely on audition and make an effective use of their developed auditory capacities. In fact, when blind individuals need to move safely in their environment, they make an extensive use of auditory information. Therefore, sound localization seems to be a superior skill in the blind. In one study, both blind individuals with and without residual vision and sighted controls localized sounds in the horizontal plane, both binaurally and monaurally (with one pinna obstructed). In the binaural condition, both groups performed equally. In the monaural condition, a subgroup of blind individuals surprisingly showed better performance than all other groups, the latter were consistently biased towards the non-obstructed ear (Lessard et al., 1998). This suggested that at least a subgroup of blind uses in an enhanced fashion the spectral cues usually treated by the pinna for sound localization. This study showed that visual deprivation does not prevent blind from forming a spatial map of their environment and in fact can perform better than the sighted. Next, scientists investigated whether neurophysiological markers could explain the behavioral gain at the neuronal level. Thus, the N1 and P3 peaks of auditory evoked potentials of blind participants localizing sounds were larger and distributed more posteriorily than in controls (Kujala et al., 1997; Roder et al., 1999; Leclerc et al., 2000). Not only blind participants were better at localizing sounds in open space but they were also able to show such superiority when monaural and binaural sound localization cues were manipulated via headphones (Weeks et al., 2000). In fact, occipital areas involved in motion processing were activated to process sounds in space. The changes in N1 amplitude suggest that the plastic adaptations in blind take place already at perceptual levels before the engagement of any high level attentional process (Leclerc et al., 2000).

Use-dependent plasticity describes the phenomenon that shapes cortical response properties when stimulations presented are behaviorally relevant. As for the blind, for whom auditory information is behaviorally important, use-dependent plasticity results in the increased efficiency for the processing of pure and frequency modulated sounds (Stevens and Weaver, 2009). This increased efficacy is translated into a finer tuning of frequencies representation in the primary auditory cortex which leads to an expansion of the tonotopic maps (Elbert et al., 2002).

Absolute pitch is a musical super-skill, which can be the result of a genetic predisposition or early musical training. Absolute pitch manifests itself with a greater variability in planum temporale asymmetry together with a left sided dominance. A higher proportion of blind musicians than sighted musicians have absolute pitch and this even if their musical training started later than the sighted musicians did. Further, the presence of this skill was correlated with increased asymmetry in the planum temporale (Hamilton et al., 2004). On the same register of spectral variation in sounds, early blind individuals show superior performance in pitch discrimination when differences between tone frequencies are small and vary with short inter-tone intervals (Gougoux et al., 2004). The superior performance in pitch discrimination, found in early but not in late blind, suggests that plastic changes are likely when visual deprivation happens early in life (Gougoux et al., 2004; Noppeney, 2007).

Auditory processing is not only based on processing of spectral cues or pitch. What about other types of sounds like voices, which blind individuals use to identify persons instead of faces as for the sighted? In fMRI, different sounds (vocal and non-vocal) induced responses in visual cortex in blind individuals but not in the sighted. But when compared to sighted and late blind, only congenitally blind subjects showed strong activation of the superior temporal sulcus (selective area for voice processing) for voice-specific processing, and this correlated with the performance in the voice discrimination task (Gougoux et al., 2009). This last result is actually in contrast with occipital involvement in the blind but in line with intra-modal changes in the auditory cortex of the blind under the experience-dependent hypothesis (Elbert et al., 2002; Stevens and Weaver, 2009).

Cross-modal plasticity in the occipital cortex for the processing of sounds leads to superior spatial localization and auditory motion processing in the blind. In fact they show involvement of the visual dorsal pathway for these auditory tasks (Weeks et al., 2000; Voss et al., 2004, 2006, 2008; Gougoux et al., 2005). Functional imaging showed occipital activations in sound-space processing providing neural correlates for the better localization in blind subjects. Early blind participants with superior monaural sound localizing abilities exhibited an increased PET signal in the occipital cortex while it was reduced in sighted controls (Gougoux et al., 2005).

Differences between early and late blind individuals seem to converge and diverge according to the experimental paradigm. They can perform better than sighted in sound localization in peri-personal and far space (Voss et al., 2004) if the cues are subtle as for the peripheral positions (monaural cues) or the far space positions (distance). For frontal binaural sound localization, they have equal performance. At the cortical level, superior performance in sound localization was correlated with occipital areas activations, although not the same areas were involved for early and late blind (Voss et al., 2008). Adaptive plasticity depends, for sound localization, on the time of onset of visual deprivation. Proficient early blind recruit left striate and bilateral extra-striate areas, correlated with the superior level of performance. Late blind, on the other hand, show activations restricted to right occipito-temporal areas. A sub group of early blind with poor performance show ventral extra-striate activations. These differential activations for monaural sound localization among early blind may be due to a different sensory-dependent experience, while the homogeneity in the late blind group with different areas involved may point to the limits of adaptive plasticity when sensory deprivation onset happens later in life, after the end of the critical period for the development of sensory systems.

Compensatory plasticity has been explored in early and late blind, for several tasks, such as pure and complex tones, pitch variation and sound-source discrimination, voice processing, sound localization in peri-personal and far space. However, the question arises about what happens for alternative behavioral paradigms. One of them investigated way finding in congenitally and late blind in a realistic maze (Fortin et al., 2008). Blind individuals in both groups who performed better than the sighted in route learning exhibited an increased hippocampus volume, a brain structure important for spatial navigation (Fortin et al., 2008; Chebat et al., 2011).

Another approach to study cortical activation in blind subjects is to use the neuromagnetic auditory steady-state response (ASSR) to amplitude-modulated tones. ASSR is an oscillatory response sensitive to the stimulation rhythms (Lazzouni et al., 2010, 2012). It was used to track stimulation frequencies and related neuronal activity in the normal auditory cortex. Responses follow a pattern where their spectral content is dominated by clear and large spectral peaks at the stimulation frequencies. The use of the ASSR to assess cross-modal reorganization in the visual cortex of the blind is based on the assumption that under visual deprivation, functional reorganization of the auditory response in occipital cortex would be tagged to the stimulation frequencies as in the auditory cortex. We carried out such a study to examine the nature of the activations to ASSR in blind and sighted subjects. Sighted controls showed stronger responses in auditory cortex compared to occipital responses. Blind individuals, on the other hand, showed equal spectral responses in auditory and occipital areas. When cortical sources of neuromagnetic signals were localized in the blind and sighted, variable source activities appeared. As in preceding studies where early blind individuals were split into two groups, namely, good performers with occipital recruitment and poor performers with less occipital recruitment (Voss et al., 2006, 2008), the ASSR response in a subgroup of blind individuals was found in primary visual areas. The surprising result was that a subgroup of sighted controls also showed occipital activations to the processing of amplitude modulated tones. This can possibly be explained by another study (see below) wherein we showed that in sighted controls, visual areas activations could be induced by rapid bottom-up mechanism. The participants carried out the task with eyes closed, wearing night masks for several minutes. In blind, ASSR functional reorganization in the occipital cortex is more likely to be mediated through cortico-cortical connections unmasked between auditory and visual areas. These results provide further evidence that visual deprivation early in life alters brain function to process auditory stimuli involving core visual areas and object vision areas.

## What about tactile, odor and taste processing in the blind?

Initial studies focused on most extensively used modalities in the blind namely touch and audition. Following studies explored the remaining sensory modalities such as taste, odor processing not to forget high level cognitive processes (Kupers and Ptito, 2014).

## Tactile modality

Although researchers investigated tactile perception performance in the blind in early 20th century, pioneering studies came with contradictory results, sometimes blind had superior performance and some others equal performance for tactile perception capabilities compared to sighted controls. Later on, on one hand, no superiority was found in tactile processing, blind and sighted showing similar performance using orientation discrimination tasks to better assess the differences in perception (Lechelt, 1988) and similar accuracy on shape matching (Heller, 1989). On the other hand, other studies confirmed the compensatory hypothesis with superior performance in the blind on tactile spatial tasks (Jones, 1975). Since then, with the development of brain imaging techniques, we have gained insight in tactile processing in the blind. In a PET study, Wanet-Defalque et al. (1988) were the first to show increased occipital metabolic activity in the blind to non-visual tasks (auditory and tactile). Reduced thresholds for tactile processing were also found (Sterr et al., 1998) and occipital cortex activation in tactile processing was confirmed (Sadato et al., 1996) as in Wanet-Defalque et al. (1988). Such visual areas activations were also found in fMRI for active tactile tasks (Burton et al., 2002; Gizewski et al., 2003; Stilla et al., 2008). Behavioral studies confirmed superiority in tactile acuity in the blind for texture discrimination (Alary et al., 2009; Sathian and Stilla, 2010). Recently, Wong and his collaborators were able to tease apart whether increased tactile acuity comes because of visual deprivation or is it extensive reliance on the sense of touch that enhances tactile spatial acuity. Indeed, spatial acuity performance in the blind and not in sighted was better for the tested fingers than for the lips (Wong et al., 2011a). In the same study, proficient Braille readers, outperformed non-readers on preferred index finger and were better with the preferred index than the opposite one. These results are in favor of the tactile experience hypothesis rather than the visual deprivation hypothesis (Sathian and Stilla, 2010).

## Odor and taste

Odor and taste processing were assessed later on, in the context of visual deprivation and the findings are informative despite the fact that studies are less numerous than for tactile and auditory processing. On the perceptual level, blind individuals outperformed sighted in free-identification, categorization and discrimination of odors, showing a better access to semantic information (Cuevas et al., 2009). On the cortical level, simple odor detection task elicited group differences between blind and sighted controls. Congenitally blind activated primary and secondary olfactory areas in addition to strong activations of occipital cortex areas (Kupers et al., 2011); reorganization is in fact possible for odor processing. Recently, an fMRI experiment showed robust right ventral occipital cortex activations to be highly correlated with superior performance in odor processing in early blind for discrimination or categorization of fruit and flower odors. The blind show a clear dissociation with verbal control task activations clearly left lateralized in ventral lateral occipital complex. Sighted controls in this case show the same lateralized right-olfactory and left-verbal pattern but all over their brain with no or little occipital activations (Renier et al., 2013). Fewer studies explored taste differences between blind and sighted and recent results show increased thresholds in taste detection and identification in the blind (Gagnon et al., 2013).

## Blindness, blindfolding and training

Cross-modal plasticity in the blind depends on time of onset of visual deprivation. In early onset blindness the brain is strongly rearranged and changes are more pronounced (Noppeney, 2007). Late onset blindness gives rise to plastic changes as well; however the behavioral gain is less and the networks involving visual areas in the processing non-visual information are fewer (Heller, 1989; Veraart et al., 1990; Büchel et al., 1998; Burton, 2003; Voss et al., 2004).

Blindfolding sighted subjects for short periods offers another model to study plasticity after sensory deprivation. Transient short-term deprivation gives evidence that plastic changes and functional reorganization are possible in adulthood, long after the critical period (Boroojerdi et al., 2000; Kauffman et al., 2002; Facchini and Aglioti, 2003; Merabet et al., 2004; Ramos-Estebanez et al., 2007; Merabet et al., 2008; Wong et al., 2011b). Blindfolding paradigms are very useful and add much to understand rapid plastic changes in the brain in a controlled way. Even short-term visual deprivation of 45 min was sufficient to induce cortical plastic changes. One phenomenon of particular interest in this context are phosphenes, retinotopic localized flashes of light, perceived without external visual stimulation. They are considered as markers of excitablity changes of visual neurons and can be induced by TMS. Forty-five minutes of light deprivation significantly lowered TMS thresholds needed to elicit phosphene perception (Boroojerdi et al., 2000).

Increase in tactile spatial acuity was possible after 90 min in complete darkness (Facchini and Aglioti, 2003). When blindfolded sighted subjects carried out tactile tasks after a short period of visual deprivation, occipital activations were observed (Pascual-Leone and Hamilton, 2001; Merabet et al., 2008). After 2 h and a half of visual deprivation, subthreshold TMS on the occipital cortex elicited a phosphene perception, only when a simulataneous subthreshold somatosensory stimulation was presented on the same side. As a control, before blindfold the same subthreshold TMS alone didn’t induce any phosphene perception (Ramos-Estebanez et al., 2007).

**Figure 1:**
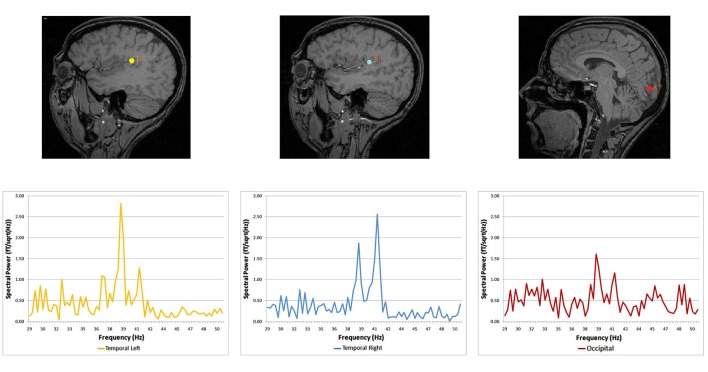
**Source locations and corresponding spectral power for the dichotic condition**. Dipolar source positions related to the ASSR are represented, co-registered on the anatomical scan of a given participant (sagittal view). Left and right auditory sources are shown for the dichotic condition, together with a source in the occipital cortex. Corresponding spectral responses are represented for each dipolar source—left and right temporal areas and the occipital area (Lazzouni et al., 2012).

Spectral analysis of cortical auditory responses tagged to specific frequency stimulation is a practical mean to track plastic changes after short-term visual deprivation. The use of blindfolds was extended several hours and auditory and visual responses recorded every 2 h using magneto-encephalography and the ASSR. Auditory cortex responded to dichotic stimulation with the stronger response being in the hemisphere contralateral to stimulated ear. The pattern of stronger contralateral cortical response to the stimulated ear is already known in the auditory system (Tesche et al., 1995; Ross et al., 2000; Pratt et al., 2009; Lazzouni et al., 2010). Visual response to amplitude-modulated tones was tagged with exact stimulation frequencies (Figure 1) in accordance with the auditory response pattern (Lazzouni et al., 2012).

The use of vision substitution devices in the blind combine the effects of training on the use of the device and early or late visual deprivation. Imaging techniques help investigate these combined effects. As such, training on the sensory substitution device can lead to metabolic changes in occipital areas in the blind (Ptito et al., 2005). Sighted controls trained blindfolded were able to show the same performance but not the same activations (Kupers and Ptito, 2014). The sensory training with a vision substitution device can be paralleled to the sensory-dependent experience in early blind with sound localization or Braille reading which results in a better performance on such processes and occipital activations (Wong et al., 2011a). Plastic changes in the blind can even induce subjective perceptions after unmasking of silent cortico-cotical pathways. In the following example, blind subjects were trained with a tongue display unit to perform visual tasks. After training, they reported subjective perceptions on their tongue, when stimulated over the occipital cortex with TMS. Since subjective perceptions are absent before training, existing connections between parietal and visual cortex may be reinforced and strengthened to induce such sensations. The hypothesis of cortico-cortical pathway strengthening may explain the induction of tactile sensations in the fingers of blind Braille readers (Kupers et al., 2006; Ptito et al., 2008). When congenitally blind individuals are trained to process soundscapes, sounds of visual objects through a visual-to-auditory-sensory substitution device, they activate areas with specialized function as the word form area or the extra striate body area in the absence of vision and for information hardly accessed by touch as for other body shapes (Striem-Amit et al., 2012a; Striem-Amit and Amedi, 2014).

## Plasticity mechanisms

Several mechanisms underlie cortical plasticity, given the complex development of the human brain. Early sensory experience shapes the brain and the functional organization of its different systems is integrated into a coherent whole. It is obvious that sensory deprivation will induce changes at different levels of brain development, structural, neurochemical and functional.

## Cortico-cortical pathways

**Figure 2:**
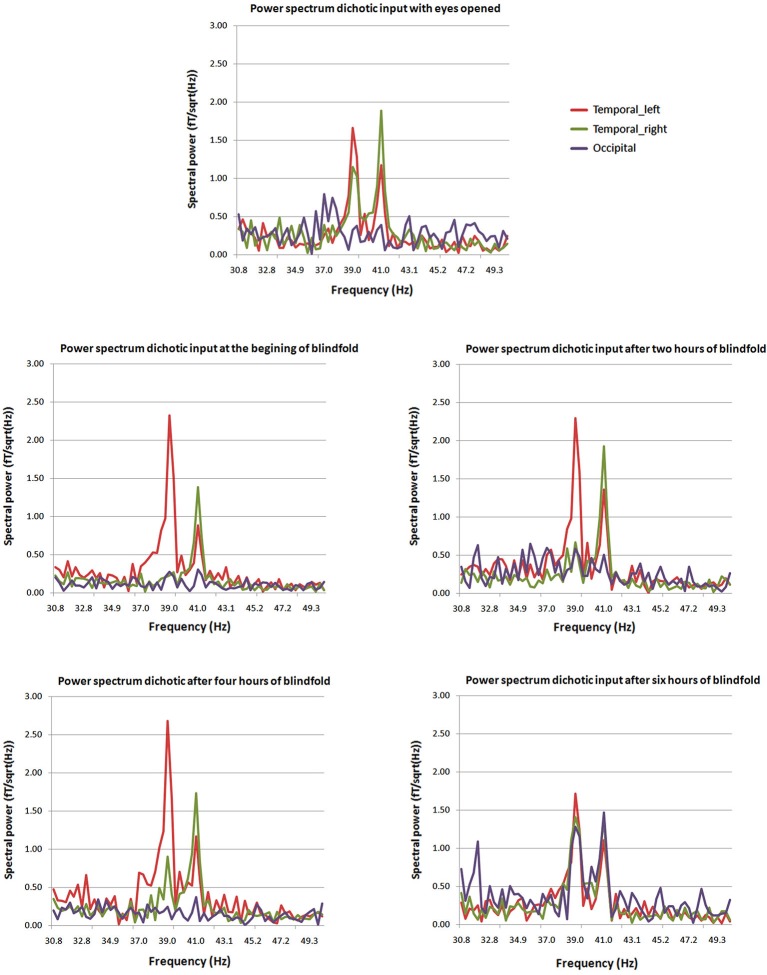
**Power spectra at different times from the beginning of blindfolding**. The two peaks at 39 and 41 Hz characteristic of the dichotic response are clearly present in the power spectrum for the posterior source (purple) after 6 h of blindfolding and are not seen clearly in previous measures. Mean average spectral peaks for the regular ASSR with eyes opened for comparison purposes are presented at the top of the figure (Lazzouni et al., 2012).

Visual areas, recruited to process cross-modal sensory information, if they are involved in higher cognitive processes need to have access to auditory and somatosensory information. Three possible pathways convey non-visual inputs to deafferented visual cortex: (1) the sub-cortical pathway; (2) the heteromodal pathway; and (3) the cortico-cortical pathways. These connections exist but they are inhibited, then are unmasked when the visual cortex is deprived from its sensory inputs (Pascual-Leone et al., 2005). More recent findings confirm cortico-cortical pathways through which cross-modal stimulations are mediated to the occipital cortex of early blind (Klinge et al., 2010). Different dynamic causal models including A1, V1 and the MGN were compared in an auditory-fMRI paradigm with congenitally blind participants. The strongest connection coefficients related to the auditory task were found between A1 and V1 (Klinge et al., 2010). Abundant anatomical equivalents to these functional connections found in animals between A1 and V1 can constitute the anatomical neural correlate of such cross-modal reorganization (Innocenti et al., 1988; Kahn et al., 2000; Falchier et al., 2002; Karlen et al., 2006; Beer et al., 2011). On the short-term deprivation time scale, visual cortex responses to amplitude-modulated tones exhibited an auditory like behavior, responding to the specific frequencies of dichotic stimulation, after few hours of visual deprivation. After few hours, the most likely mechanism to explain rapid reorganization of visual response to stimulation rhythms is the cortico-cortical pathway (Pascual-Leone et al., 2005; Lazzouni et al., 2012; Figure 2). While most participants had a visual response tagged to stimulation frequency at the end of the 6 h of blindfolding time, few individuals showed a faster reorganization of the occipital response to amplitude modulated tones at the beginning of visual deprivation more likely through sub-cortical connections with a complete takeover of the auditory response by the visual cortex. The cortico-cortical pathways can play an important role in cross-modal plasticity in the blind for more basic auditory and tactile sensory processing and explain the behavioral gain (Gougoux et al., 2004; Wittenberg et al., 2004; Alary et al., 2009; Voss and Zatorre, 2012). Therefore, we cannot set aside the role of multimodal higher order areas when it comes to more complex stimulation as for tactile or sound objects recognition and localization or the use of vision substitution devices (Amedi et al., 2002, 2010; Renier et al., 2010; Striem-Amit et al., 2012b; Striem-Amit and Amedi, 2014), beyond basic sensory processing.

## Neurochemical and structural changes

**Figure 3:**
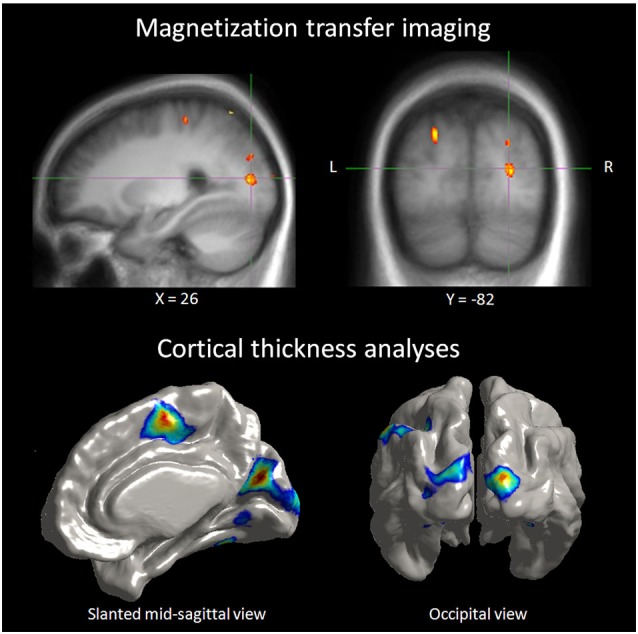
**Correlation between neuroanatomical and behavioral measures in blind individuals**. Top panel: illustration depicting a right extrastriate visual area where the MT ratio (an indirect measure of myelin content) in blind individuals was found to be predictive of behavioral performance in a pitch discrimination task. Bottom panel: illustration depicting occipital regions where cortical thickness in blind individuals was positively correlated to the same performance measures of the pitch discrimination (Voss and Zatorre, 2012).

Neurochemical changes assessed with magnetic resonance spectroscopy in the blind showed higher levels in the occipital cortex of creatine, a marker of energy usage in neurons and glia. Increased levels of creatine may explain chronic metabolic adaptation in the visual cortex (Weaver et al., 2013). It is well known that the metabolic demand in the occipital cortex of the blind is elevated compared to sighted (Veraart et al., 1990). Higher levels of choline in the occipital cortex of the blind are difficult to explain in blindness, as choline is a marker of membrane breakdown in brain disease. Increased concentration of myo-inostol relates to increased number or size of glial cells, and this change was found in the blinds’ occipital cortex. Bilateral and unilateral deprivation is accompanied with down-regulation of GABA-ergic network. Weaver and collaborators found indeed lower levels of GABA in the occipital cortex of their blind subjects (Weaver et al., 2013). All these neurochemical changes were present and at the same time, the proportion of gray, white matters and tissue within occipital and precentral voxels did not differ between the blind and sighted controls. Therefore, metabolic changes do not relate to macroscopic differences in brain tissue composition among groups. Morphological differences exist actually in congenitally, late blind and sighted. Congenitally blind show reduced primary visual areas surface extent despite increased thickness in peri-calacrine areas, while late blind show cortical thinning in visual cortex with no surface extent reduction (Park et al., 2009). Increased cortical thickness in early visual cortex and other structural changes in cortical thickness associated with early and late visual deprivation are among the changes observed. In different visual areas of the blind, cortical thickness, was correlated with superior task-specific performance in discrimination of pitch and melody (Figure 3), giving yet another evidence of anatomical representation of adaptive compensatory plasticity (Voss and Zatorre, 2012) and its functional role. Changes in anatomy were not restricted to occipital cortex, as early blind exhibit reduced volume of the posterior part of hippocampus while the head of the hippocampus size is larger in early and late blind, which was associated with higher performance in a way-finding task (Chebat et al., 2007a; Fortin et al., 2008).

## Connectivity networks and plasticity in the occipital cortex

Most task-based experiments confirmed the compensatory hypothesis, superior performance and visual cortex involvement in sensory processing of cross-modal stimulation. Imaging resting-state in the blind helps explore functional connectivity and coupling between brain regions and the way sensory deprivation alters intrinsic neuronal interactions.

**Figure 4:**
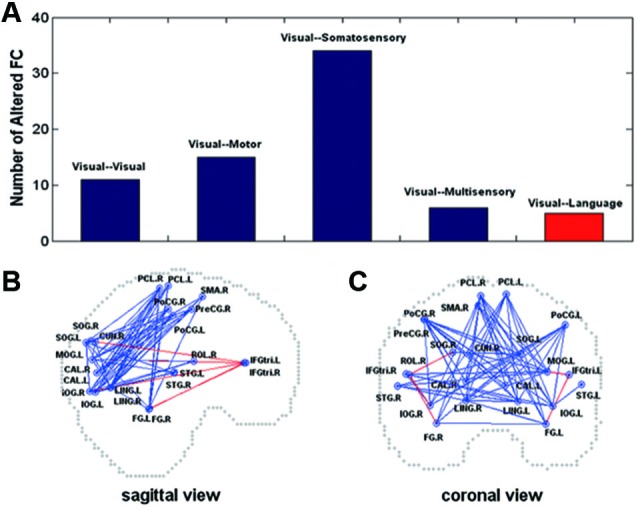
**Altered functional connectivity in the early blind (A) shown on sagittal (B) and coronal (C) views**. In **(A)**, the *y*-axis indicates the number of the pairs with altered functional connectivity. In **(B)** and **(C)**, the dots represent the centroids of each brain region. The blue color represents decreased functional connectivity and the red color denotes increased functional connectivity in the early blind. FC = functional connectivity (Liu et al., 2007).

Magneto-encephalography imaging at rest can assess frequency-specific intrinsic neuronal interactions in the blind, in such case correlations increase in delta and gamma band oscillations across visual cortex. This indicates strong functional coupling in the occipital cortex (Hawellek et al., 2013). Brain oscillations are involved in cognitive processes such as memory, attention and integrative functions (Basar et al., 2001), processes that are altered by early blindness. Cortical networks functional architecture is mirrored by resting-state activity dynamics. As long as functional architecture is determined by genes and shaped by experience, patterns of spontaneous activity may serve as a “covert internal model for perception and action” (Singer, 2013). Within the context of this statement, most prevalent features of function architecture are present in resting state and allow the transition to a large number of realisable brain states, such as sensory and cognitive functions (Heisz et al., 2012; Singer, 2013). Functional connectivity in the whole brain assessed by comparing correlation coefficients calculated between each pair of brain regions is informative during resting-state. The resting-state connectivity networks derived from these correlations are compared between blind and sighted (Liu et al., 2007). Blind subjects demonstrate decreased functional connectivity within occipital areas. Somatosensory, frontal motor and temporal multisensory areas connectivity with occipital cortex decrease as well. Such reduction in connectivity in the blind compared to sighted controls may be in agreement with a general loss hypothesis. However, this is not the end of the story, because blind individuals with early introduction and extensive practice of Braille reading increase and thereby show a modulation in functional connectivity among brain regions (occipital, somatosensory). Moreover, early onset blind individuals show strong functional connectivity between occipital areas and frontal language areas, especially for a subgroup of proficient Braille readers (Figure 4). It is hypothesized that the general loss mechanism plays a dominant role and that compensatory plasticity develops in childhood and improves with extensive sensory practice (Liu et al., 2007). Resting-state functional connectivity in congenitally blind is maintained between higher visual areas and the primary somatosensory cortex and in the dorsal stream. It was decreased between primary somatosensory cortex and early visual areas and the decrease was more pronounced in the ventral stream. When blind individuals execute a non-haptic form recognition task using a tongue display unit after extensive training on the device, the ventral stream is activated although it was not activated before training (Ptito et al., 2012). This confirms that altered sensory experience and training can improve adaptive plasticity (experience-dependent adaptation) (Wong et al., 2011a) by changing the intrinsic functional connectivity in the visually deprived brain. The organization of function of the visual cortex, in terms of relations between primary and higher order visual areas, and in terms of the relation of both with other sensory and motor systems and to the ventral and dorsal streams, seem to depend differently on visual experience (Qin et al., 2013).

## Conclusions

The generally superior performance of early and late blind individuals correlated with functional task-specific activation of visual cortex in sensory and cognitive processes confirms the compensatory plasticity hypothesis. Blind individuals make an efficient use of their spared modalities within a highly malleable brain. However, superior performance in the blind is not the dominant state. Poorer performance and absence of visual activations in the early or late blind would fall under the general loss hypothesis. However, even blind individuals who do not out-perform generally do as well as the sighted. The absence of vision does not prevent calibration of senses or formation of a map of the surrounding space.

Experimental conditions aimed to disentangle sensory deprivation compensatory effects, the influence of the age of visual loss or the importance of experience and training in the sensory loss context, are constantly reviewed and adapted, with the help of imaging and brain stimulation techniques. Early blindness gives a greater opportunity for more pronounced plastic changes, in performance and occipital cortex recruitment through cortico-cortical pathways. Plastic changes also depend on early sensory-dependent experience and extensive training, as for example for Braille reading and music. However, it is also clear that not all early blind individuals are equal. As seen throughout the manuscript, early and late blind visual deprivation do not always result in the same functional reorganization or performance improvement. While early blind individuals show more often enhanced behavioral performance and occipital involvement in cross-modal tasks, late blind on the other hand show normal behavior and less occipital areas activations. Some of them do not benefit from compensatory plasticity to develop superior skills. They lose sight later, for a shorter duration and have reduced non-visual sensory experience compared to early and congenitally blind. Moreover, cortical plasticity is also possible in adulthood and gives similar chances to perform better on sensory and cognitive tasks; it seems to rely on similar mechanisms but involves a recruitment of visual areas to a lesser extent. New and promising currents in resting-state networks show that even if intrinsic connectivity is altered in the congenitally deprived brain, it is still susceptible to change by different early experiences, on which depends the organization of the visual cortex. Still, the potential for change is important and compensatory adaptation can take place despite a dominant general loss state. Possible changes in default connectivity in the sensory deprived brain allow for neuro-rehabilitation by extensive practice and/or training to reinforce experience-dependent adaptation.

## Conflict of interest statement

The authors declare that the research was conducted in the absence of any commercial or financial relationships that could be construed as a potential conflict of interest. The reviewer, Théoret declares that, despite being affiliated with the same institution as the authors, the review process was handled objectively.
